# Routine monitoring and assessment of adults living with HIV: results of the British HIV Association (BHIVA) national audit 2015

**DOI:** 10.1186/s12879-017-2708-y

**Published:** 2017-09-13

**Authors:** A. Molloy, H. Curtis, F. Burns, A. Freedman, on behalf of the BHIVA Audit and Standards Sub-Committee

**Affiliations:** 1 Royal Free London NHS Foundation Trust, Pond Street, London, NW3 2QG United Kingdom; 2 British HIV Association, c/o Mediscript Ltd, 1 Mountview Court, 310 Friern Barnet Lane, London, N20 0LD United Kingdom; 3 Royal Free London NHS Foundation Trust AND Research Department of Infection & Population Health, University College London, Pond Street, London, NW3 2QG United Kingdom; 4 British HIV Association AND Clinical Reader in Infectious Diseases and Honorary consultant Physician in General Medicine & Infectious Diseases at University Hospital of Wales, Heath Park, Cardiff, CF14 4XW United Kingdom

**Keywords:** HIV, Diagnostics, Prevention

## Abstract

**Background:**

The clinical care of people living with HIV changed fundamentally as a result of the development of effective antiretroviral therapy (ART). HIV infection is now a long-term treatable condition. We report a national audit to assess adherence to British HIV Association guidelines for the routine investigation and monitoring of adult HIV-1-infected individuals.

**Methods:**

All UK sites known as providers of adult HIV outpatient services were invited to complete a case-note review and a brief survey of local clinic practices. Participating sites were asked to randomly select 50–100 adults, who attended for specialist HIV care during 2014 and/or 2015. Each site collected data electronically using a self-audit spreadsheet tool. This included demographic details (gender, ethnicity, HIV exposure, and age) and whether 22 standardised and pre-defined clinical audited outcomes had been recorded.

**Results:**

Data were collected on 8258 adults from 123 sites, representing approximately 10% of people living with HIV reported in public health surveillance as attending UK HIV services. Sexual health screening was provided within 96.4% of HIV services, cervical cytology and influenza vaccination within 71.4% of HIV services. There was wide variation in resistance testing across sites. Only 44.9% of patients on ART had a documented 10-year CVD risk within the past three years and fracture risk had been assessed within the past three years for only 16.7% patients aged over 50 years.

**Conclusions:**

There was high participation in the national audit and good practice was identified in some areas. However improvements can be made in monitoring of cardiovascular risk, bone and sexual health.

## Background

In 2014, an estimated 103,700 people were living with HIV (PLWH) in the UK, with 85,489 seen for HIV care during the year [1, 2]. Improvements in treatments for HIV mean that life expectancy for PLWH has increased dramatically [3]. However with longer life expectancy we are seeing an increasing proportion of people living with co-morbidities including cardiovascular disease, metabolic complications, and malignancy [4, 5]. As such, to ensure optimal health outcomes for our patient cohort, primary and secondary health promotion and disease prevention extending beyond the virological and immunological control of HIV is required. The British HIV Association (BHIVA) produces guidelines for the management of HIV infection, in the UK. The process used by the BHIVA has been accredited by the National Institute for Health and Clinical Excellence (NICE). Guidelines are drafted by BHIVA Writing Groups and then placed on the BHIVA website for consultation by all interested parties. The *BHIVA guidelines for routine investigation and monitoring of adult HIV-1 infected individuals 2011* recognised the paradigm shift in the treatment of HIV infection and provided guidance on the appropriate monitoring and assessment of adults living with HIV [6]. These guidelines present a consensus regarding the standard assessment and investigation of HIV infection from the time of diagnosis and describe the appropriate monitoring of HIV-positive individuals both on and off ART.

In 2015, as part of the national audit programme of the British HIV Association (BHIVA), we audited adherence to the BHIVA guideline on monitoring and assessment of HIV infected individuals, and where relevant, immunisation guidelines. This article reports our findings.

## Methods

All UK sites known to BHIVA as providers of adult HIV services were invited to complete a case-note review and a brief survey of local clinic practices. During June to August 2015 participating sites were asked to randomly select from the local list, 50–100 adults (aged 16 or over), who attended for specialist HIV care during 2014 and/or 2015. The selection was random and was not stratified. The number of cases was selected to ensure maximum participation of sites with varying patient populations. Data at each site were collected electronically using a self-audit spreadsheet tool. This included demographic details (gender, ethnicity, HIV exposure, and age) and whether audited outcomes as listed in Table 1 had been recorded. Some of these were modified from outcomes as specified in guidelines for ease of assessment. To allow for varying appointment dates, monitoring/procedures recommended as “annual” were interpreted as within 425 days (14 months). Similarly six monthly was taken to mean within 243 days (8 months), except in the case of HIV viral load testing where the guideline recommendation is to test 3–6 monthly i.e. within six months, rather than at 6 month intervals. As some HIV clinics do not provide influenza vaccination or cervical cytology, information was sought as to whether individuals had been advised to obtain these elsewhere.

**Table 1:** Demographics of sample

All	8258	100.0%
Sex		
Male	5482	66.4%
Female	2763	33.5%
Transgendered/ transgendering	9	0.1%
Not stated	4	0.0%
Ethnicity		
White	4853	58.8%
Black-African	2592	31.4%
Other	733	8.9%
Not recorded/not stated	80	1.0%
HIV Exposure		
Heterosexual	4320	52.3%
MSM	3550	43.0%
IDU	153	1.9%
Other	99	1.2%
Not recorded/not stated	136	1.6%
Age		
16–29	674	8.2%
30–49	5001	60.6%
50–69	2396	29.0
70+	172	2.1%
Not stated	15	0.2%

This is the first national audit of adherence to the BHIVA monitoring and assessment guidelines. Data were compared to the targets set in the guidelines where applicable. Individual participating sites received their results via the self-audit spreadsheet tool and could compare them with national results which were presented at the BHIVA Autumn conference 2015 [7]. Statistical analysis was performed using Microsoft Excel. Further analyses assessing the effect of adjusting for patient mix on site-level outcomes and summarising outcomes into meaningful groups and presenting results as a visual dashboard have been performed and reported elsewhere [8].

## Results

Case-note review data was provided for 8258 patients from 123 sites, but only 112 services completed the survey of clinic practices. The 8258 individuals included in the case-note review represent approximately 10% of PLWH reported in public health surveillance as attending UK HIV services [2]. Two thirds were male, just over half (52.3%) acquired their HIV heterosexually, the majority were of white ethnicity (58.8%) and 60.6% were in the 30–49 year age group (see Table 1). 62.0% (5119/8258) of patients had been reviewed within three months up to audit data extraction in June–August 2015 and 96.5% (7971/8258) had been reviewed within 1 year. While it is difficult to perform a direct comparison, the characteristics of our study population are similar to the reported demographic data on people living with HIV in the UK [1].

Table 2 shows the proportion of individuals achieving the audit standards for all audited recommendations.

**Table 2:** Clinical monitoring standard examined and performance

Clinical Monitoring requirement audited	Specified target in guidelines	Proportion of cases with documentation of meeting audit standard % (n/N)
Whether a baseline HIV resistance test had been done or sample stored for later testing	Patients with a genotypic resistance test performed within 3 months of first diagnosis (or with a stored sample available for later testing) (90%).	80.8% (6636/8258 tested, 40/8258 sample stored)
Whether HIV viral load measured within past 6 months	Patients on ART with HIV viral load measured within the last 6 months (80%).	90.1% (6660/7395)
Whether adherence assessed within past 425 days	Adherence documented within the first 3 months of starting ART (90%) and at least annually thereafter (70%).	93.4% (6908/7395)
Whether all medication recorded within past 425 days	All medication taken by patients on ART should be reviewed annually (100%).	89.0% (6584/7395)
Whether vaccinated/immune to hepatitis A	No target specified but serology recommended followed by vaccination for all non-immune at risk and/or co-infected with hepatitis B or C.	61.2% (5053/8258)
Whether hepatitis B serology recorded; whether anti-surface antibody measured within past 425 days for individuals with serology consistent with vaccination	No target specified but surface antigen (HBsAg), anti-core total antibody (anti-HBc) and anti-surface antibody (anti-HBs) testing recommended. Vaccination recommended if non-immune. Annual surface antibody titre measurement recommended in vaccine responders.	82.1% (6781/8258)
Whether hepatitis C antibody status known	No target specified but antibody testing recommended, followed by RNA testing if antibody positive. Annual re-testing recommended for antibody negative men who have sex with men (MSM) or injecting drug users (IDU).	96.6% (7979/8258)
Whether CVD risk assessed, within past 3 years if on ART, ever if not on ART	10-year cardiovascular disease (CVD) risk calculated within 1 year of first presentation (70%), and within the last 3 years if taking ART (70%).	44.9% (3318/7395) on ART 32.3% (279/863)
Whether smoking status recorded within past two years; if a smoker, whether offered a cessation service.	Smoking history documented in the last 2 years (90%) and if a smoker offered referral to a cessation service (90%).	65.9% (5445/8258) 45.2% (862/1905) offered cessation
Whether blood pressure recorded within past 425 days	Blood pressure (BP) recorded in the last year (90%).	85.5% (7058/8258)
Whether glucose measurement recorded within past 425 days	No target specified but recommended yearly or 3–6-monthly if on ART.	77% (6359/8258)
Whether lipid profile recorded within past 425 days	No target specified but recommended yearly or 6–12-monthly if on ART.	83.2% (6869/8258)
Whether liver function test (LFT) assessed within past 425 days	No target specified but recommended yearly or 3–6 monthly if on ART.	97% (8013/8258)
Whether estimated glomerular filtration rate (eGFR) assessed within past 425 days	No target specified but recommended yearly or 3–6 monthly if on ART.	95.5% (7887/8258)
Whether urinalysis or urine protein/creatinine (uP/C) checked within past 425 days, or 243 if receiving tenofovir	No target specified but urinalysis and uP/C recommended annually, with 3–6-monthly urinalysis if receiving tenofovir.	73.7% (2050/2781) 74.8% (4098/5477) receiving tenofovir
Whether flu vaccination had been done or record made of advice to obtain this from general practitioner (GP) within past year: as audit was conducted in summer this fully covered the preceding season	No target specified in monitoring guidelines but vaccination history recommended as part of regular clinical review. Vaccination guidelines specify: offer annual influenza vaccination to all HIV-infected persons (target 95%) [14].	21.1% (1744/8258) vaccination given 36.2% (2993/8258) advice given
Whether sexual health screen offered within past 425 days	No target specified but recommended to offer sexual health screen 12-monthly, or more frequently if identified risks.	65.7% (5424/8258)
Whether syphilis serology had been done within past 243 days	No target specified but recommended 3–6-monthly at each routine visit for MSM and 12-monthly for others	63.0% (5201/8258)
Whether cervical cytology had been done or record made of advice to obtain elsewhere within past 425 days, females only	No target specified but recommended 12-monthly.	53.2% (1471/2763)
Whether bone mineral density measured, individuals aged >70 and on ART only	No target specified but recommended in all men aged 70 years and all women aged 65 years.	17.4% (29/167)
Whether fracture risk assessed within past 3 years, individuals aged >50 only	No target specified but recommended 3-yearly if aged over 50 years.	16.7% (430/2568)
Outcome: whether vaccinated against pneumococcus, CD4 > 200 only	No target specified in monitoring guidelines but vaccination history recommended as part of regular clinical review. Vaccination guidelines recommend pneumococcus vaccination if CD4 > 200, and consideration of vaccination at lower CD4.	26.4% (2082/7877)

*ART* antiretroviral therapy

### Baseline resistance testing

While the majority of audited cases met the national standard for baseline resistance testing (see Table 1), almost 1 in 5 (19.2%, *n* = 1586/8258) individuals did not. However baseline resistance testing was recorded as “not possible” for 47.3% (750/1586); 7.1% (112/1586) had neither been tested nor had a sample stored; and for 45.4% (720/1586) it was not known whether this had been done or the question was not answered.

### Antiretroviral treatment

Of 89.5% (7395/8258) audited individuals who were on ART, 90.1% (6660/7395) had a HIV viral load measurement within the past 6 months, exceeding the guideline target of 80%, and 93.4% (6908/7395) had had ART adherence assessed in the last year, which outperformed the target of 70%. However, only 89.0% (6584/7395) had all other medications recorded within the last year, despite a target of 100% to avoid drug-drug interactions.

### Viral hepatitis

Among audited individuals, 61.2% (5053/8258) were hepatitis A vaccinated, immune, or seropositive, 11.9% (*n* = 983/8258) were seronegative, 0.02% (2/8258) equivocal, and for 26.9% (2220/8258) it was not known whether this had been done or the question was not answered.

In terms of hepatitis B, 82.1% (6781/8258) individuals had serology fully reported (hepatitis B surface antigen HBsAg, hepatitis B core antibody (anti-HBc) and hepatitis B surface antibody (anti-HBs)); 0.9% (72/8258) were HBsAg positive with incomplete antibody status; 10.2% (841/8258) were HBsAg negative with incomplete antibody status; 6.8% (564/8258) had unknown HBsAg status or the question was not answered. Among 306 chronic hepatitis B infected (HBsAg+) individuals, 7.8% (24/306) were apparently unvaccinated and seronegative for hepatitis A. Among 3605 individuals whose status was consistent with vaccination (anti-HBs+, anti-HBc- and HBsAg-), 67.0% (2416) had had an annual anti-HBs measurement.

Hepatitis C antibody (anti-HCV) status was negative for 91.3% (7539/8258) individuals, positive for 5.3% (439/8258), equivocal for 0% (1), not known or the question was not answered for 3.4% (279/8258). Among the 7539 seronegative individuals, 65.4% (4928) had had an annual re-test including 74.1% (2423/3270) men who have sex with men (MSM) and 61.8% (21/34) injecting drug users (IDU), groups in whom this is recommended. Hepatitis C RNA (ribonucleic acid) testing had been done for 91.1% (400/439) anti-HCV positive patients. Data on HCV genotyping were not collected.

### Cardiovascular health

Only 44.9% (3318/7395) of patients on ART had a documented 10-year CVD risk within the past three years. Of those not on ART, 32.3% (279/863) had 10-year CVD risk recorded at any time, despite targets of 70% for both groups. ART status was unclear for 18 patients. Among 1582 patients aged >50 years on ART, with no documentation of established CVD, almost half (48.7%; 770) had CVD risk calculation recorded within the last 3 years.

Smoking status had been documented within the past two years for 65.9% (5445/8258) audited patients, well below the target of 90%. Only 45.2% (862/1905) of current smokers had been offered a cessation service although this is recommended [9]. Current smokers were not more likely to have CVD risk calculated than ex or never smokers.

### Sexual and reproductive health

An annual sexual health screen was recorded as offered for 65.7% (5424/8258) of all patients, including 72.7% (2581/3550) MSM, and 60.8% (2627/4320) heterosexuals. Syphilis serology was recorded within the past eight months (243 days) for 63.0% (5201/8258) of all patients, 73.4% (2604/3550) MSM, and 55.3% (2390/4320) heterosexuals.

Out of 2763 women, 53.2% (1471) had annual cervical cytology done and 21.9% (604) had been advised to attend a GP or sexual health clinic for this. The self-audit spreadsheet tool did not provide an option for women ineligible for cervical cytology. Contraception was reported not relevant for 31.7% (877/2763) of women. It had been discussed for 63.0% (1188/1886) women for whom it was relevant.

### Bone health

Fracture risk had been assessed within the past three years for only 16.7% (430/2568) patients aged over 50 years. Bone mineral density had been measured in 17.4% (29/167) individuals aged over 70 years and receiving ART. For simplicity, the spreadsheet recorded this from age 70 for both sexes although guidelines recommend it from age 65 in females.

### Variation in monitoring between participating sites

There was wide variation in resistance testing across sites. 27% (33/123) sites met the target, having a recorded resistance test or stored sample for >90% of audited individuals while 27% (33/123) sites achieved this outcome for <75% of audited individuals, including 4.9% (6/123) sites who did so for <60%.

There was variation between sites in the proportion of patients for whom HBsAg status was not known, and for 8 sites this exceeded 20%. There was wide variation across sites in provision of influenza vaccination with 16/123 sites having administered or advised obtaining influenza vaccination at their general practitioner (GP) or Pharmacy for fewer than 10% of patients and 17/123 sites for more than 90% of patients.

There was wide variation in CVD risk calculation between sites; of 122 sites submitting data for patients receiving ART, 21.3% (26) met the 70% target for CVD risk recording within three years but 27.9% (34) achieved this for fewer than 20% of patients. There was also variation across sites for fracture risk assessment.

### Monitoring frequency

Most survey respondents routinely reviewed patients who were stable on antiretroviral therapy every 6 months, when HIV viral load (VL) was also checked. CD4 counts were less frequently measured (see Table 3). Sexual health screening was provided within 96.4% (108/123) of HIV services, cervical cytology within 71.4%(80/123) of HIV services and influenza vaccination within 71.4%(80/123) of services.

**Table 3:** Frequency of routine monitoring for individuals virologically stable^a^ on ART (112 clinical services)

	Clinical review	HIV viral load measurement	CD4 count measurement
3 monthly	4 (3.6%)	6 (5.4%)	5 (4.5%)
4 monthly	17 (15.5%)	24 (21.4%)	16 (14.3%)
6 monthly	74 (66.1%)	78 (69.6%)	42 (37.5%)
Yearly	10 (8.9%)	1 (0.9%)	45 (40.2%)
Other or not answered	7 (6.3%)	3 (2.7%)	4 (3.6%)

^a^virologically stable = HIV viral load undetectable on standard assay (<50 copies/ml)

## Discussion

There was high participation in the national audit and the data showed good practice in some areas. Monitoring of ART was performed well; the proportions of individuals with viral load measurement and adherence assessment exceeded the expected standard. CD4 counts were less frequently measured in line with recent evidence on CD4 count monitoring frequency [9]. The proportion of individuals with all medications recorded was high. Sexual health screening was performed well, perhaps due to the availability of this service in the same or adjacent clinics. Overall recording of hepatitis serology was high but with wide variation between sites. There was also wide variation in influenza vaccination, recording of baseline resistance testing, and monitoring of CVD risk factors and bone health. Some sites reported very low rates of baseline resistance testing though it is possible that this may reflect patients diagnosed and commenced on ART before routine resistance testing was available or recommended [10, 11].

Cardiovascular disease and smoking are major causes of morbidity and mortality in people living with HIV [4, 12]. However, low recorded rates of monitoring of cardiovascular health were noted and smoking status was not reported for one in seven patients, and less than half of current smokers were offered cessation support. The growing population of older patients living with HIV were also overlooked in terms of bone health, with very low rates of fracture risk assessment and bone mineral density recording. It is not clear if low recorded rates of monitoring of bone density was due to a lack of availability of bone densitometry measurement.

At some sites, a significant proportion of patients had unknown HBsAg status and many patients remain susceptible to hepatitis A, including those with documented chronic hepatitis B infection, for whom hepatitis A could be more serious. There was very low reported coverage of influenza and pneumococcal vaccination, perhaps indicating an issue for patients when responsibility and funding is shared between primary and secondary care.

### Limitations

Participating sites were asked to select individuals randomly from those attending during 2014 and/or 2015, but it is possible that more recent, and perhaps more regular, attenders were over-sampled as suggested by the fact that 62.0% of individuals had been reviewed within three months and 96.5% within one year preceding the audit. This may have led to over-estimation of rates of performance and recording of routine interventions.

There was also difficulty in interpreting some targets in the BHIVA guidelines. For example the 90% target for baseline resistance testing (or sample storage) is applicable for new incident diagnoses but less clear for individuals established in HIV care. As baseline resistance has ongoing clinical relevance, it is important this information remains accessible, including when individuals transfer care between clinics. We considered the target not met (80.8% individuals having recorded test or sample stored) although only 1.4% were known to be untested and some of these may have started ART before resistance testing was in use. As for all the monitoring outcomes some findings may reflect incomplete recording or reporting rather than performance.

## Conclusion

Clinical services should review and develop systems to prompt both performance and recording of recommended interventions. Some sites have an ‘annual review’ system or electronic reminders for monitoring and, if adopted more widely, these may improve compliance with the monitoring guidelines. There may be a perception that primary care is responsible for certain monitoring and services such as cardiovascular risk and providing most vaccinations. Hence greater clarity from commissioners about where responsibility lies in these areas, particularly in what the forthcoming tariffs for HIV care cover, will help HIV services focus on areas they are responsible for. Improved communication between HIV and primary care services would streamline and improve care for people living with HIV. Clinical services should also develop improved strategies to care for older patients living with HIV [13].

## Abbreviations

anti-HBc: Hepatitis B core antibody
anti-HBs: Hepatitis B surface antibody
anti-HCV: Hepatitis C antibody
ART: Antiretroviral therapy
BHIVA: British HIV Association
BP: Blood pressure
CVD: Cardiovascular disease
eGFR: Estimated glomerular filtration rate
GP: General practitioner
GU: Genitourinary
HBsAg: Hepatitis B surface antigen
HIV: Human immunodeficiency virus
IDU: Injecting drug users
LFT: Liver function tests
MSM: Men who have sex with men
NHS: National health service
PLWH: People living with HIV
RNA: Ribonucleic acid
UK: United Kingdom
uP/C: Urine protein/creatinine
